# Uremic Pancolitis in an Adult Patient with Newly Diagnosed, Rapidly Progressive Crescentic Immunoglobulin A Nephropathy

**DOI:** 10.7759/cureus.3826

**Published:** 2019-01-04

**Authors:** Hector H Gonzalez, Meaghan McMahon, Angel E Sanchez, Jennifer G Foster, Ira Lazar

**Affiliations:** 1 Internal Medicine, Florida Atlantic University Charles E. Schmidt College of Medicine, Boca Raton, USA; 2 Miscellaneous, Florida Atlantic University Charles E. Schmidt College of Medicine, Boca Raton, USA; 3 Nephrology, Florida Atlantic University Charles E. Schmidt College of Medicine, Boca Raton, USA

**Keywords:** colitis, iga nephropathy, uremia

## Abstract

Uremic gastroenteropathy is a well-accepted but less often described sequelae of an underlying renal disease. With the advent of modern dialysis treatments, rarer manifestations, such as pancolitis, may go overlooked in the evaluation, pursuing more common diagnoses. The underlying pathophysiology of uremic gastroenteropathy is not completely understood; however, several underlying mechanisms have been identified to play a role. Here, we present an exceptionally rare case of uremic pancolitis in a Hispanic male who presented with clinical, imaging, and pathological findings consistent with newly diagnosed, rapidly progressive crescentic IgA nephropathy (IgAN).

## Introduction

Uremic pancolitis is a rare manifestation of an impairment in the intestinal barrier due to underlying risk factors and intestinal insults. Local pro-inflammatory cytokines (interleukin 1 (IL-1), Tumor necrosis factor-a (TNF-a), IL-6, IL-13) lead to the propensity for barrier degeneration [1]. The insult in renal disease, particularly end-stage renal disease, results in uremic toxins, which transverse the intestinal barriers into circulation and have the potential to affect many organ systems. The subsequent disease process that ensues as a cause of uremia includes pericarditis, colitis, and encephalopathy.

IgA nephropathy (IgAN) is the most common form of glomerulonephropathy, accounting for an overall population incidence of 2.5 cases per 100,000 [2]. IgAN has a propensity for progression to end-stage renal disease in as many as 50% of cases within 20 years [3]. The pathophysiology of IgAN consists of the abnormal glycosylation of IgA-deficient galactose residues of unknown etiology, which serve to induce a host immune system cascade [4-5]. This cascade results in the deposition of complement C3, C4, and IgA within the mesangium. The typical initial presentation consists of gross hematuria, microscopic hematuria, or nephrotic syndrome. The clinical presentation of acute renal failure in the setting of IgAN is rare, with several suggested etiologies in the literature to date. One such cause is the tubular occlusion of red blood cell casts that arise in the context of massive hematuria to which the glomerulus is exposed [6]. The other proposed mechanism is through the deposition of crescents within the glomerulus, which was evident upon our patient's renal biopsy.

Despite the rarity of rapidly progressive crescentic IgAN, in as few as 5% of all IgAN cases, its diagnosis confers a very poor prognosis [3]. Clinicians should remain cognizant to the implications of this rare entity in the hopes of providing timely diagnosis and treatment modalities. We present a rare case of a Hispanic male with newly diagnosed, rapidly progressive crescentic IgAN, presenting with uremic pancolitis.

## Case presentation

A 40-year-old man with no past medical history presented to the emergency department with weakness, generalized abdominal pain, nausea, and intractable vomiting of one-week duration. He also endorsed multiple episodes of loose, non-bloody bowel movements. He denied any fevers/chills, hematochezia, melena, voiding difficulty, dysuria, hematuria, or flank pain. Of note, the patient denied any prior history of abdominal pain, changes in bowel habits, or underlying family history of gastrointestinal or renal disease. The patient, however, stated that he had not seen a physician in the past 18 years.

On physical examination, his temperature was 36.8°C, heart rate 69 bpm, respiratory rate 16 br/min, blood pressure 147/102 mmHg, and oxygen saturation 100% on room air. The patient appeared diaphoretic and in moderate distress. The abdomen was soft, diffusely tender, no distension/guarding /rigidity, and normoactive bowel sounds, costovertebral angle (CVA) tenderness, and Murphy sign negative.

Laboratory data revealed a white blood cell count of 6100 K/mcl, hemoglobin 5.9 g/dL, hematocrit 18%, and platelet count 240,000 K/mcl. The basic metabolic panel revealed sodium of 130 mmol/L, potassium 4.7 mmol/L, chloride 95 mmol/L, CO_2_ 24 mmol/L, blood urea nitrogen (BUN) >150 mg/dL, creatinine 26.9 mg/dL, anion gap 21, glucose 87 mg/dL, and serum calcium 6.5 mg/dL. Liver function studies and lipase were within normal limits. The fecal occult blood test (FOBT) was negative. Anemia workup showed normal iron, low total iron binding capacity (TIBC), high ferritin, normal B12, and low reticulocyte count. Arterial blood gas showed pH 7.35, pCO_2_ 18, pO_2_ 149. Lactic acid was 0.4 mmol/L, erythrocyte sedimentation rate (ESR), and C-reactive protein (CRP) were normal. Urinalysis showed microscopic hematuria, proteinuria, and a urine protein to creatinine ratio of 2.5.

**Figure 1:**
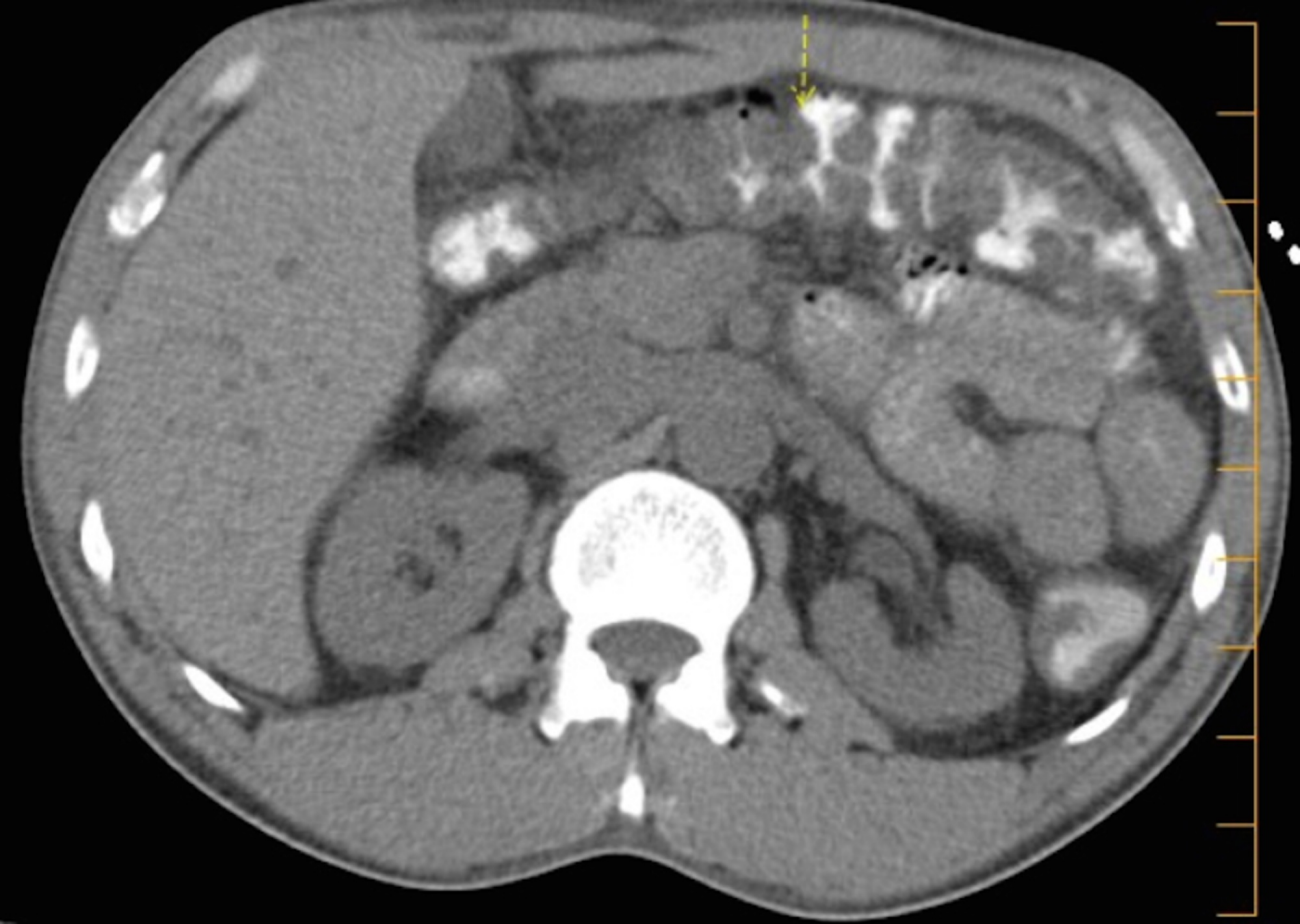
A computed tomography scan showing bowel wall thickening with “thumbprinting” and surrounding mesenteric inflammatory change

**Figure 2:**
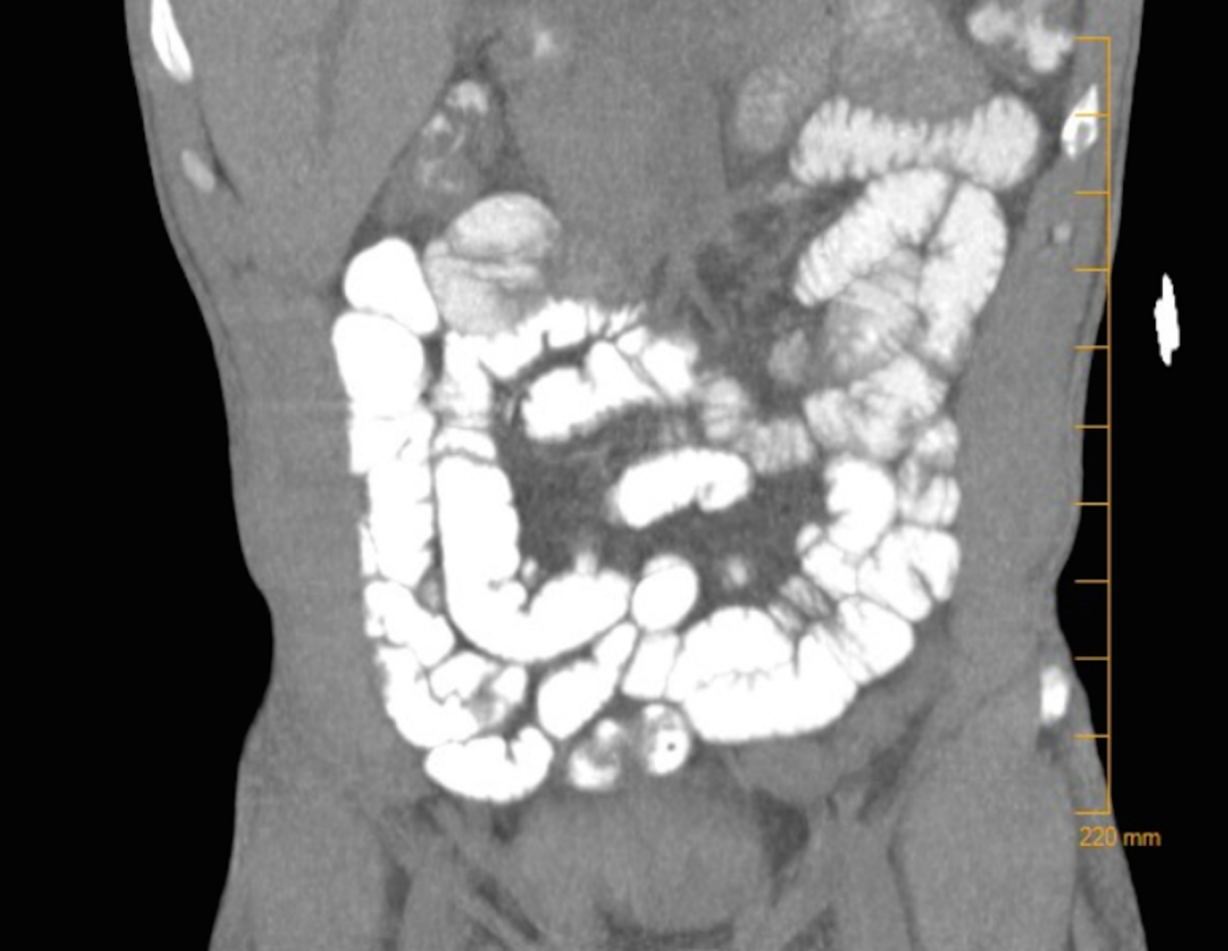
A computed tomography scan showing diffuse bowel wall thickening from the terminal ileum throughout the entire colon

Imaging revealed renal ultrasound with an increased echogenicity of both kidneys without atrophy or hydronephrosis. Computed tomography (CT) abdomen/pelvis revealed diffuse bowel wall thickening from the terminal ileum throughout the entire colon with thumbprinting noted, surrounding mesenteric inflammatory changes extending to the rectum, and 9 cm kidneys (Figures 1-2). There was no visualized lymphadenopathy or retroperitoneal mass on CT.

Subsequently, the patient was    transfused two units of packed red blood cells, given analgesics, and placed on intravenous fluids at 100 cc/hour. Nephrology and gastroenterology were consulted. On hospital Day 2, the patient was noted to have persistent anion gap metabolic acidosis, uremia, and hyperkalemia with hyperphosphatemia. The electrocardiogram obtained showed a sinus rhythm with no acute ST wave changes. The patient was given D50 with insulin administration. He subsequently underwent hemodialysis with ultrafiltration. On hospital Day 3, the patient endorsed a significant improvement in his abdominal pain with the ability to pass formed stool. He was placed on oral amlodipine, sevelamer, and sodium bicarbonate.

Further laboratory testing showed that parathyroid hormone was elevated, consistent with secondary hyperparathyroidism. Hepatitis B, C, and HIV were negative. Serum complement studies demonstrated a low level of C3 but normal C4. Double-stranded deoxyribonucleic acid (DNA), immunoglobulin G (IgG), rheumatoid factor, cytoplasmic antineutrophil cytoplasmic antibodies (c-ANCA), perinuclear-ANCA (p-ANCA), and glomerular basement antibody were negative. There was no monoclonal gammopathy on serum protein electrophoresis (SPEP)/urine protein electrophoresis (UPEP). Fecal leukocyte, stool culture, ova/parasite, Clostridium difficile (C. diff) toxin assay, and Escherichia coli (E. coli) O157:H7 Ag testing were negative. The peripheral smear was unremarkable and demonstrated no schistocytes.

**Figure 3:**
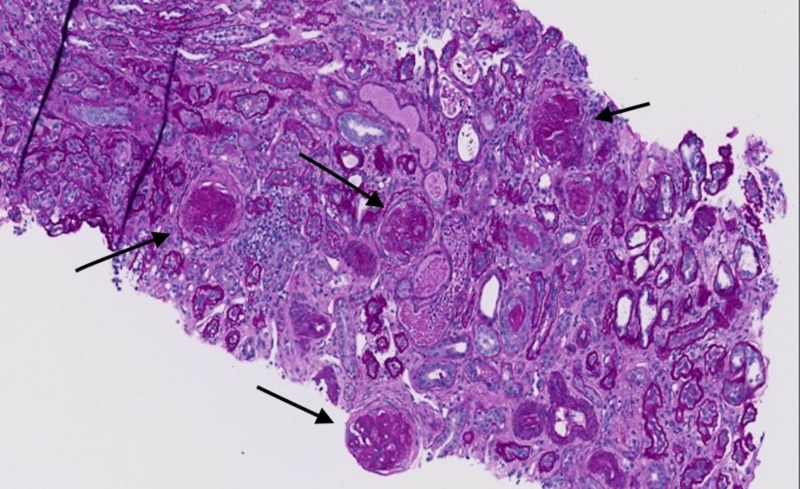
Sclerotic glomeruli (arrows) with interstitial fibrosis and tubular atrophy

**Figure 4:**
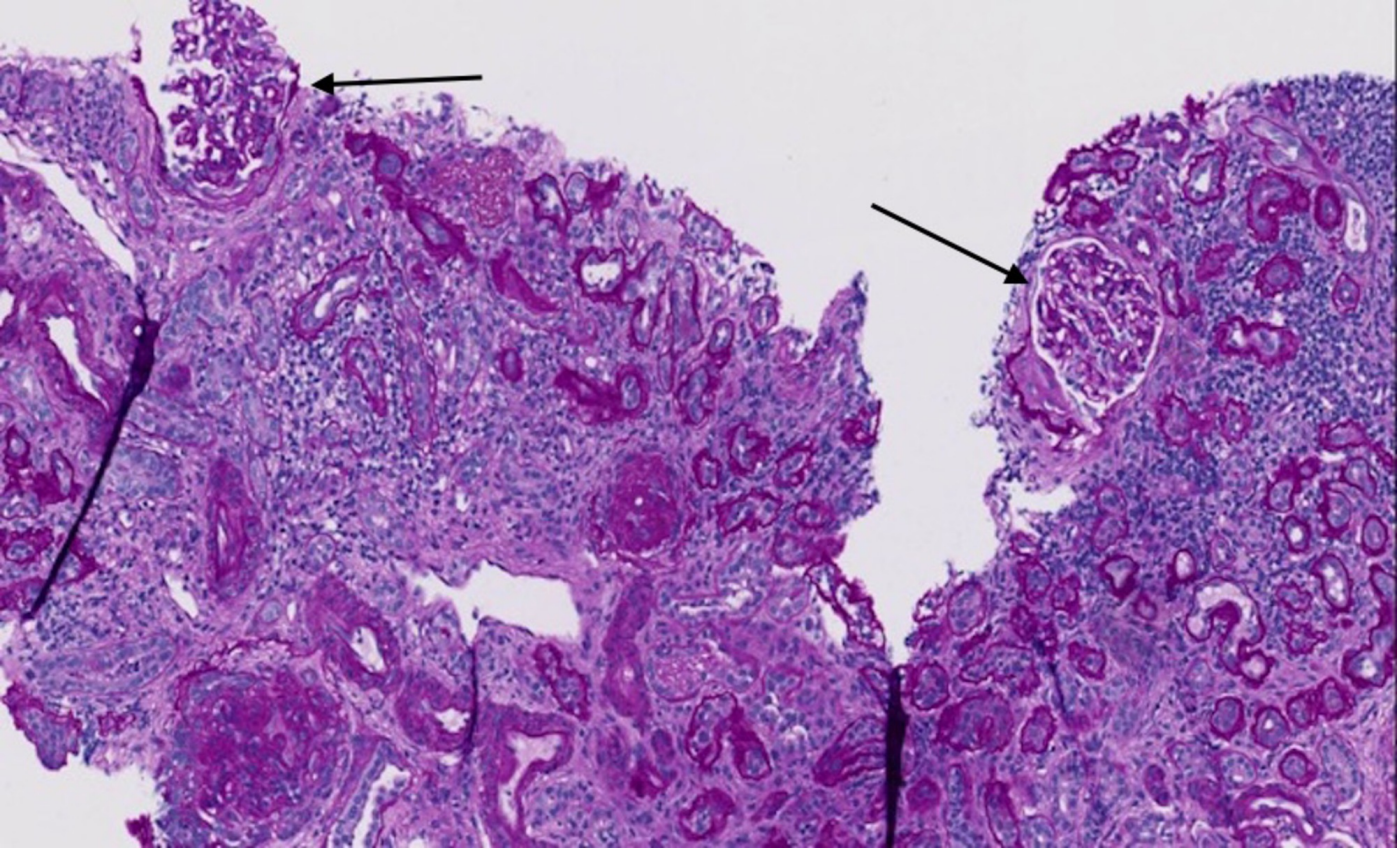
Periodic acid–Schiff stain showing two glomeruli with open capillaries (arrows)

Throughout subsequent days, the patient had complete resolution of abdominal complaints, however, despite providing supportive therapies with intravenous fluids, red blood cell transfusions, and hemodialysis, the patient continued to have a worsening of his BUN and creatinine. A renal biopsy was obtained to evaluate for underlying glomerular disease, which revealed several glomeruli with fibrous-fibrocellular crescents, widespread prominent interstitial fibrosis, and tubular atrophy, with 90% glomerulosclerosis consistent with IgAN (Figures 3-4).

**Figure 5:**
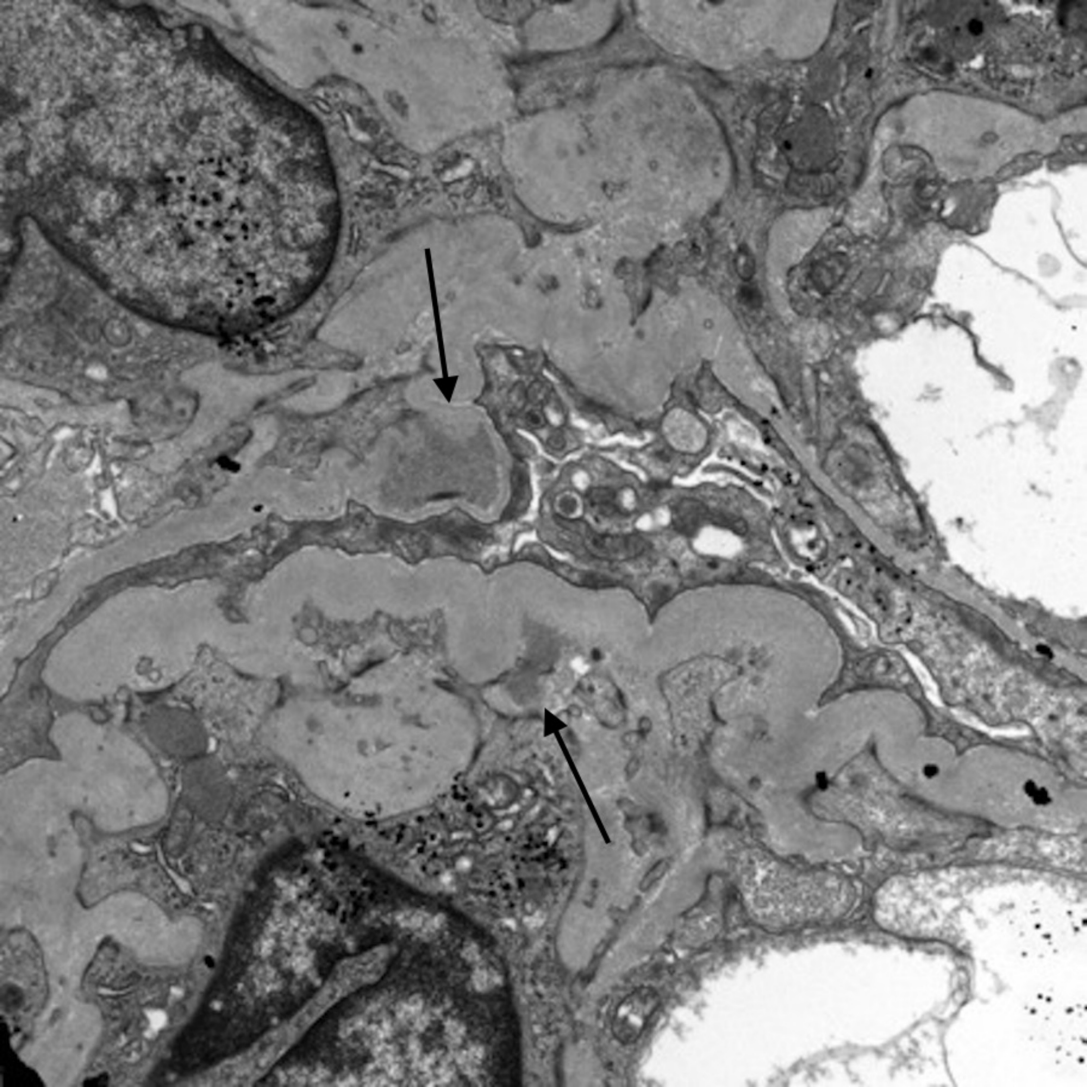
Scattered mesangial and intramembranous dense deposits

Electron microscopy revealed scattered mesangial and intramembranous dense deposits (Figure 5).

**Figure 6:**
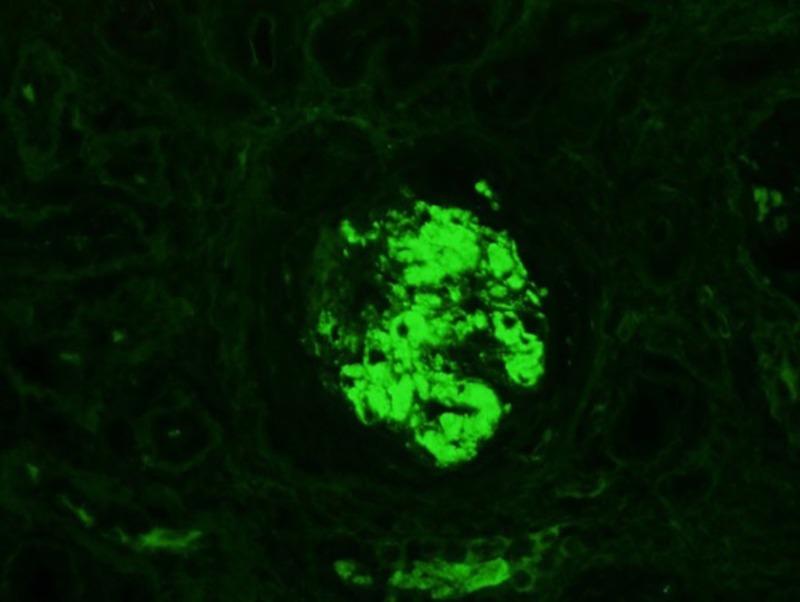
IgA staining noted in a sclerotic glomerulus indicating glomerular sclerosis secondary to IgA nephropathy IgA: immunoglobulin A

**Figure 7:**
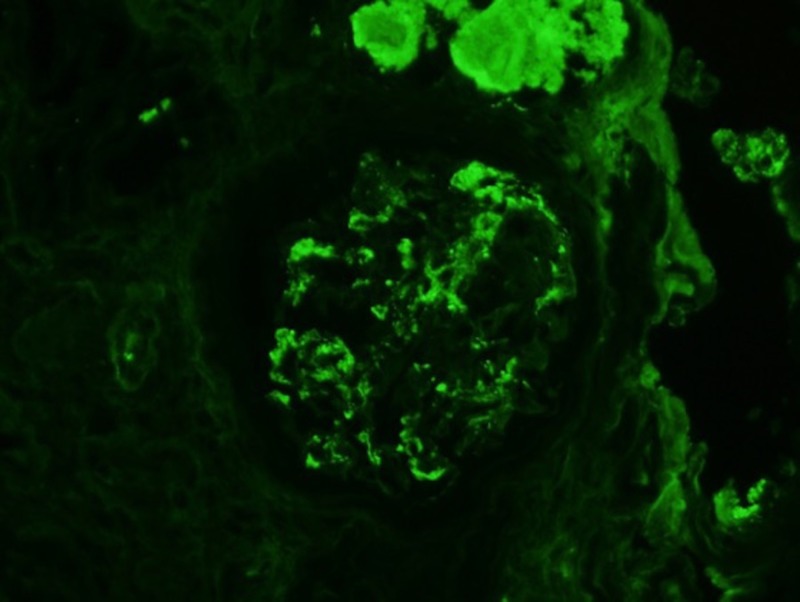
Mesangial IgA deposits seen in a glomerulus with open capillaries. Bright enhancements above glomerulus indicating casts. IgA: immunoglobulin A

Immunofluorescence performed showed mesangial IgA deposits seen in a glomerulus with open capillaries, and IgA staining in a sclerotic glomerular with bright enhancements indicating casts (Figures 6-7).

It was determined by nephrology that he would need long-term dialysis. He underwent left upper extremity radiocephalic arteriovenous (AV) fistula creation and right internal jugular tunneled catheter placement for outpatient hemodialysis access. He was discharged with instructions to follow up in the outpatient department with dialysis.

## Discussion

The imaging finding of pancolitis can often be misdiagnosed as arising due to ulcerative or pseudomembranous colitis due to the inflammatory pattern. In the context of gastrointestinal symptoms, renal failure, and anemia, the diagnosis of hemolytic uremic syndrome (HUS) may be premature. Due to the vast differential, uremia-associated colitis is often ignored among clinician, with other etiologies, such as inflammatory bowel disease and infectious colitis, higher on their differential. Our patient had an undiagnosed history of IgA nephropathy, which contributed to his presentation of renal failure. There are no cases identified in the American literature that have previously found uremia leading to pancolitis. Recent studies attribute the destruction of colonic epithelial integrity and changes in gut microbiology to uremia contributing to systemic inflammatory responses [7]. Toxicity rising in the setting of uremic translocation and other toxins have been linked in prior studies to contribute toward gastrointestinal manifestations [8].

Studies have been conducted aimed at identifying certain markers, such as Nrf2 (transcription factor nuclear factor erythroid 2- related factor 2), which contribute to chronic inflammation in the setting of chronic kidney disease [1]. This factor has a central role of serving to upregulate enzymes to aid as antioxidants in the setting of stressors and inflammation throughout the body. During renal failure, the massive inflammatory host response, oxidative stress, and toxins serve to deplete Nrf2, which leads to a propensity for compromised intestinal barriers [1]. In terms of management, certain modalities aimed at decreasing the levels of urea should be taken to prevent further sequelae of uremia. Interventions such as emergent dialysis, avoiding excessive fluid resuscitation, a high-fiber diet, and the addition of activated charcoal may aid in ameliorating the systemic detrimental effects of uremia. Our patient had a complete resolution of all gastrointestinal manifestations of uremia with dialysis treatment. It is clear that additional comparative diagnostic and treatment studies are necessary in the future to establish appropriate management.

Recognition and awareness of the presence of IgAN in the Hispanic population are critical to timely diagnosis and management. The typical presenting symptoms of IgAN include gross hematuria, microscopic hematuria, and proteinuria. The differential diagnosis for patients presenting with low serum complement levels includes vasculitis, infection, hepatitis C, and atheroembolic kidney disease. Our patient was found to have a low level of C3 but normal C4. The complement levels in patients with IgAN have been documented to vary, with some patients not showing any abnormality, which hinders an accurate prognostic interpretation. The prognosis with IgAN is related to the level of GFR, the degree of proteinuria, and hypertension. Less commonly, this condition may manifest as rapidly progressive glomerulonephritis with acute onset renal failure, hypertension, and edema. IgA nephropathy has classically been associated with East Asian and Caucasian ethnicities. Based on a literature review, few studies have addressed the epidemiology of IgAN in Hispanic populations. A large series of renal biopsies from Peru noted lower prevalence rates of IgAN, which accounted for only 0.9% of all glomerular lesions over a 10-year period in Lima [9]. Another study performed at the University of California, San Francisco (UCSF) showed that 3% of all patients with IgAN were Hispanic [10].

Patients with rapid progressive crescentic IgAN have histopathological renal biopsy results that reveal cellular/fibrous crescents, fibrinoid necrosis, and arteriolar damage [4]. These unique findings often times resemble those seen in patients with vasculitis. The presence of antineutrophil cytoplasmic antibody (ANCA) has been reported among a subset of those with IgAN, with no identifiable correlation to disease process to date. The presence of ANCA in IgAN has been seen both in the presence and absence of renal biopsy-proven crescents. Henoch-Schonlein purpura (HSP), a small vessel vasculitis, has been documented in the literature to manifest in patients diagnosed with IgAN. Due to the documented relationship between HSP and IgAN, there has been a suspicion that the crescents present in IgAN could be a manifestation of renal vasculitis [4]. However, further in-depth studies need to be conducted to derive a conclusion regarding the questionable relationship and how this influences the current management.

The treatment of IgAN has been divided into two subgroups: immunosuppressive and non-immunosuppressive therapies [10]. However, there is no established guideline for appropriate treatment modalities in IgAN. Patients are treated with angiotensin-converting enzyme (ACE) inhibitors or angiotensin II receptor blockers (ARB) for blood pressure control and proteinuria [5]. To date, there are trials regarding the treatment of IgA nephropathy, including the TESTING randomized clinical trial that compared Solu-Medrol to placebo. This trial found that in patients with IgAN and proteinuria of 1 g/d or greater, oral steroids were associated with increased infections [11]. There were some patients that showed benefit, however, the trial ended prematurely, which prevents us from extrapolating a definitive treatment benefit. Another recent trial studied the role of rituximab, showing that peripheral B cell counts decreased, however, there was no effect on under-glycosylated IgA1, immune complexes, nor severity of disease [12]. Although IgAN is less commonly seen in the Hispanic population, we must consider this diagnosis in a Hispanic patient presenting with acute renal failure, hematuria, and proteinuria. The early diagnosis of rapidly progressive IgAN will ensure improved disease course, prognosis, and clinical outcomes.

## Conclusions

This case presents an exceptionally rare presentation of uremic pancolitis in a Hispanic male who presented with newly diagnosed rapidly progressive crescentic IgA nephropathy (IgAN). Despite the fact that rapidly progressive crescentic IgAN represents less than 5% of all cases of IgAN, clinicians should keep this in their differential diagnosis when confronted with pancolitis. Obtaining a thorough history, physical examination, and appropriate diagnostic workup are necessary to deliver timely treatment. Further studies are needed to help guide formal diagnostic criteria and treatment modalities.
